# Therapeutic Potential of Isoxazole–(Iso)oxazole Hybrids: Three Decades of Research

**DOI:** 10.3390/ijms26157082

**Published:** 2025-07-23

**Authors:** Urszula Bąchor, Marcin Mączyński, Aleksandra Sochacka-Ćwikła

**Affiliations:** Department of Organic Chemistry and Drug Technology, Faculty of Pharmacy, Wroclaw Medical University, 50-556 Wroclaw, Poland; urszula.bachor@umw.edu.pl (U.B.); marcin.maczynski@umw.edu.pl (M.M.)

**Keywords:** oxazoles, isoxazoles, heterocyclic hybrids, biological activity

## Abstract

Heterocyclic compounds are a common subject in the field of medicinal chemistry due to their numerous pharmaceutical applications. Among these, nitrogen- and oxygen-containing five-membered heterocyclic rings, namely oxazole and isoxazole, are particularly significant, exhibiting a broad spectrum of biological activities. Molecular hybridization, the process that enables the fusion of bioactive scaffolds, is a powerful strategy for the development of novel compounds characterized by enhanced or multitarget activities. This review focuses on hybrids incorporating linked oxazole and/or isoxazole moieties (i.e., isoxazole–oxazole and isoxazole–isoxazole hybrids), drawing upon peer-reviewed research articles and international patents from 1995 to the end of 2024. The overview systematically presents the diverse biological activities reported for the isoxazole–(iso)oxazole hybrids, including anticancer, antibacterial, antitubercular, anti-inflammatory, and antidepressant effects, alongside their corresponding chemical structures. Our analysis of the literature highlights the structural versatility and therapeutic potential of this important class of heterocyclic hybrids.

## 1. Introduction

Among all known pharmaceuticals, a significant amount of them are molecules with heterocyclic moieties [1]. Heterocyclic compounds belong to molecules containing one or more rings (commonly five- or six-membered rings), of which at least one atom is a heteroatom, usually nitrogen (N), oxygen (O) or sulfur (S). The chemistry of heterocycles and their biological activities are at present extensively well-explored and constitute about sixty-five percent of the organic chemistry literature [2]. Heterocycles containing nitrogen and oxygen are extensively used in several studies on natural products and the synthesis of pharmaceutical agents [3]. The majority of the biological properties of some heterocyclic compounds, as described in the literature, can be summarized as being agents exhibiting numerous activities, such as antibacterial, anticancer, genotoxic, antimicrobial, antiviral, COX-2 inhibitor, anti-inflammatory, anti-oxidant and cytotoxic activities [4]. *N*-containing heterocyclic compounds play a pivotal role in the discipline of medicinal and organic chemistry due to their extensive range of applications in the discovery of novel pharmacologically active compounds [5]. One of the critical classes of five-membered nitrogen-containing heterocyclic compounds with one or more heteroatoms, such as sulfur, oxygen, or nitrogen, is azoles [6]. The 1,2-oxazoles (isoxazoles) and 1,3-oxazoles (oxazoles) are *N*,*O*-containing five-membered heterocycles that can be widely found in many drugs and natural products [7]. Therefore, they can be used as a main compound in the pharmaceutical industry for the synthesis of various drugs. One of the most commonly used methods to develop new compounds is molecular hybridization [8]. The formation of heterocyclic hybrids is achieved through the combination of two or more bioactive scaffolds into a novel, single molecule that exhibits potential pharmaceutical activity [9]. The structural versatility, diverse biological activities, and potential for optimizing pharmacological properties present these heterocyclic hybrids as potent tools against a spectrum of biological targets [10]. Within this perspective, a significant number of heterocyclic hybrids with different bioactive moieties have been synthesized in recent years, including, among others, isoxazole–oxazole and isoxazole–isoxazole hybrids. The resulting hybrids exhibit diverse pharmaceutical applications, utilizing anticancer [11], antibacterial [12], anti-inflammatory [13], immunosuppressive [14], antitubercular [15], antiviral [16] and antifungal [17] effects. This review highlights the growing significance of isoxazole–(iso)oxazole hybrids, demonstrating their unique chemical and biological features. An effort was made to analyze the selected compounds in terms of their biological activity and to present the chemical structures of the most active hybrids.

A key focus in modern organic synthesis is molecular hybridization. The application of this approach extends beyond the isoxazole/oxazole scaffold, having been successfully implemented across a wide range of chemical classes, including heterocycles, natural products and peptidomimetics. The chemical linking of appropriately substituted moieties, separately exhibiting biological activity, into one covalently bonded compound may result in a potential synergistic effect of their independent biological activities. This strategy exploits the synergistic advantages of different structural motifs to enhance drug-like properties, such as potency, selectivity, pharmacokinetics and safety profiles [18]. Isoxazole and oxazole rings are well known for their biological activities and favorable physicochemical properties, as demonstrated by numerous studies [19,20]. Thus, the combination of these heterocycles in a hybrid compound makes it possible for complementary binding interactions to occur within the biological target, potentially leading to increased affinity or multifunctional activity. Hybridization is powerful tool in drug discovery, precisely because of the development of multifunctional agents targeting multiple biological pathways, and it is promising in the treatment of various diseases such as cancer, infectious diseases and neurological disorders [21,22,23]. The present overview demonstrates the significant value of hybridization in the field of modern medicinal chemistry.

This review was compiled using a comprehensive search strategy that included collecting data from journals and patents published up to December 2024, sourced from databases such as SciFinder, Google Patents, ScienceDirect, Scopus, PubMed, PubChem, ResearchGate and Elsevier. The search criteria aimed to identify isoxazole–isoxazole and isoxazole–oxazole hybrids, taking into account every possibility of combining these structures. In particular, we only included the hybrids with two pharmacophores, connected directly via one covalent bond. Then, we selected compounds exhibiting biological activities and we divided the collected data for separate chapters describing different biological activity, alongside their corresponding chemical structures. The aim of this review is to demonstrate the broad spectrum of biological activities associated with isoxazole–(iso)oxazole hybrids. Consequently, the overview included data from both peer-reviewed journals and the patent literature. Although the level of validation may differ among the sources, particularly in terms of biological activity, it was essential to include the complete range of reported bioactivity. Despite these differences in reliability, especially in patent data, our intent was to highlight the diverse therapeutic potential of these hybrid compounds.

## 2. Treatment of Blood Disorders and Heart Diseases

**Figure 1:**
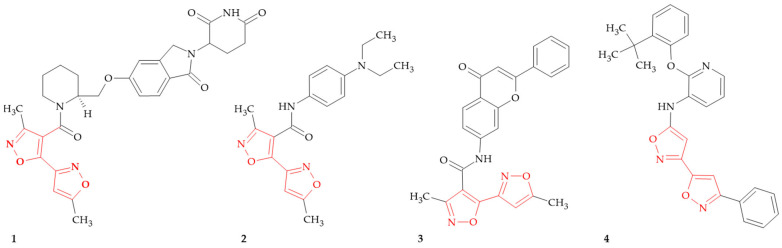
Isoxazole–isoxazole linked hybrids to treat blood and heart disorders.

Recent advances in the molecular hybridization of isoxazole rings have had a significant impact on the discovery of agents for various blood and heart disorders (Figure 1). These hybrid compounds exhibit their biological activity through three diverse mechanisms, including Widely Interspaced Zinc Motif (WIZ) degradation activity or the inhibition of the GATA binding protein 4 (GATA4) and the purinergic P2Y_1_ receptor.

### 2.1. WIZ Degradation Activity

Sickle cell disease and its variants represent the most common inherited blood disorders worldwide. In 2021, an estimated 7.74 million individuals were living with the condition, and approximately 515,000 babies were born with it that year alone [24]. This medical term includes a group of common inherited disorders with regard to a point mutation involving the gene which encodes the hemoglobin subunit β (HBB). The therapeutic induction of fetal hemoglobin (HbF) can ameliorate disease complications, and has been actively pursued [25]. The Widely Interspaced Zinc Motifs (WIZ) protein is a chromatin-associated transcription factor which localizes to promoters and enhancers. Evidence supports WIZ as a putative fetal hemoglobin (HbF) gene control factor that regulates HbF expression. To treat sickle cell disease, targeted WIZ depletion is being explored as a mechanism to re-activate HbF expression. The authors present compounds that can reduce WIZ expression levels or induce fetal hemoglobin (HbF) expression and in that way can be used to treat inherited blood disorders, e.g., hemoglobinopathies, beta-hemoglobinopathies, sickle cell disease and beta-thalassemia [26]. Compound **1** (Figure 1) was evaluated for WIZ degradation activity using a Hibit tag fusion protein assay. The data shows that the WIZ degradation activity of this compound in the WIZ HiBit assay in 293 T cells was as follows: WIZ half maximal activity concentration (AC_50_) 1.346 μM, WIZ maximum activity (A_max_) 22.2% and percentage of degradation of WIZ (100-A_max_) 77.8%.

### 2.2. GATA4 Inhibitors

The GATA binding protein 4 (GATA4) belongs to a family of transcription factors (GATA1−6) and its action is involved in the regulation of hormone response and mechanical stress as well as in cardiac repair and regeneration [27]. Moreover, GATA4 regulates myocardial gene expression by interacting with different cardiac-specific transcription factors, such as NKX2-5. The study revealed that compound **2** (Figure 1) acts as an enhancer in the GATA4 Luciferase screening assay at 10 μM concentration (% of control mean ± SD: 120 ± 0.83), and compound **3** (Figure 1) also acts as a potent enhancer in the GATA4 Luciferase screening assay at (120 ± 13) and as a potent enhancer in the NKX2-5 Luciferase screening assay (110 ± 1.5). Jumppanen and coworkers previously identified phenylisoxazole carboxamide as a hit compound which inhibited GATA4-NKX2-5 transcriptional synergy [28]. The data implies that transcription factors GATA4 and NKX2-5 directly interact with and synergistically activate several cardiac genes and stretch-induced cardiomyocyte hypertrophy [29]. The authors continued the search for the potent and selective inhibitors of GATA4-NKX2-5, and they synthesized and characterized 220 derivatives and structurally related compounds. SAR analysis revealed that the aromatic isoxazole substituent in the southern part (when the isoxazole ring is presented) regulates the inhibition of GATA4-NKX2-5 transcriptional synergy [27]. Compound **2** showed 89.24% control of mouse GATA binding protein 4 transcriptional activity in COS-1 cells upon incubation with 3 μM compound for 24 h by Luciferase assay.

### 2.3. P2Y_1_ Receptor Inhibitors

The purinergic P2Y_1_ receptor belongs to the purinergic receptors family and it plays a vital role in platelet activation, particularly in the context of thrombosis and hemostasis. The P2Y_1_ receptor is a G-protein-coupled receptor that is activated particularly by adenosine diphosphate (ADP). As the P2Y_1_ receptor is essential for the initial phase of the aggregation process, its agonists can therefore be used as hemostatic agents, promoting clot formation in patients with bleeding disorders or during surgical procedures [30]. Sutton et al. presented a synthesis of a series of novel heteroaryl compounds which are selective inhibitors of the human P2Y_1_ receptor [31]. Compounds were tested in the P2Y_1_ binding assay and are considered to be active if they exhibit a Ki value of equal to or less than 1 μM, and compound **4** (Figure 1) meets this criterion, and therefore can potentially be used in thrombotic disorders.

## 3. Treatment of Nervous System Diseases

**Figure 2:**
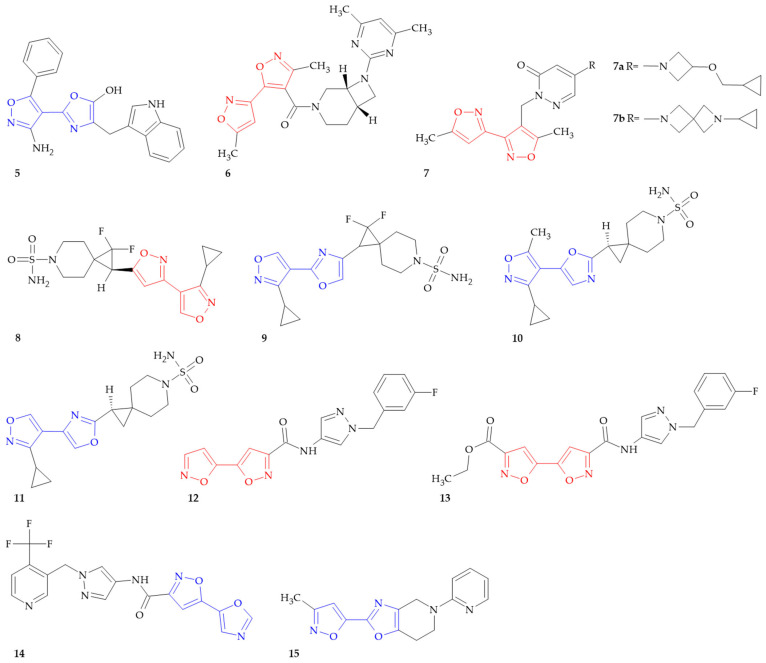
Isoxazole–(iso)oxazole hybrids to treat central nervous system disorders.

The design of isoxazole–(iso)oxazole hybrids has enhanced the treatment options for nervous system disorders, including neuropsychiatric and neurodegenerative diseases (Figure 2). These compounds exert their therapeutic effects through antidepressant and sedative activity, the inhibition of orexin receptor type 2 (OX2R) and stearoyl-CoA desaturase (SCD), or the modulation of γ-aminobutyric acid (GABA_A_) α5 receptor, the nicotinic acetylcholine receptor (nAChR) and metabotropic glutamate receptor 5 (mGlu5 receptor).

### 3.1. Antidepressant and Sedative Activity

Antidepressant and sedative agents are commonly used in the treatment of depression and anxiety disorders. Antidepressant activity refers to the modulated level of neurotransmitters such as serotonin, norepinephrine, and dopamine, which results in mood improvement [32]. In contrast, sedative activity causes a reduction in nervous system activity, which has calming effects and improves sleep maintenance [33]. The antidepressant and sedative activity of novel heterocyclic tryptophan-hybrid derivatives was investigated by Elmegeed and coworkers [34]. Compound **5** (Figure 2) showed significant antidepressant activity in the forced-swimming test at a dose of 100 mg/kg. The effect of the tested compound was more potent than the control agent L-tryptophan (100 mg/kg). Also, compound **5** produced a significant decrease in the locomotor activity of mice during a 30 min observation period at level 71% and 66% at doses of 50 and 100 mg/kg, respectively. It is evident that the sedative effect of this compound is dose-dependent.

### 3.2. OX2R Inhibitors

Orexins (hypocretins) are two neuropeptides secreted from orexin-containing neurons, mostly in the lateral hypothalamus. Orexins direct their actions by binding and activating two G-protein–coupled receptors (GPCRs), orexin receptor type 1 (OX1R) and type 2 (OX2R). Orexin/receptor pathways play important regulatory roles in many physiological processes, especially feeding behavior, sleep–wake rhythm and energy balance. Furthermore, several reports have demonstrated that orexin/receptor pathways are involved in pathological processes of neurological diseases such as narcolepsy, depression, ischemic stroke, drug addiction and Alzheimer’s disease [35]. Branstetter et al. described two series of diazabicyclic compounds as useful as orexin receptor (OR) modulators for the treatment of diseased states, disorders and conditions mediated by orexin activity, such as insomnia [36]. Compound **6** (Figure 2) acts as modulator of orexin/hypocretin receptor type 1 and 2, with values of 10 μM and 1.1 μM, respectively.

### 3.3. GABA_A_ α5 Receptor PAMs

γ-Aminobutyric acid (GABA) is the primary inhibitory neurotransmitter in the central nervous system and it is a main coordinator of brain activity. The inhibitory action of GABA is mediated by two types of receptors: GABA_A_ receptors and GABA_B_ receptors [37]. GABA_A_R is one of the key drug targets in the treatment of different neuropsychiatric disorders such as insomnia, epilepsy, anxiety, autism or eating disorders. Cecere et al. presented the synthesis of compounds useful for treatment or prevention, and mainly for GABA_A_ α5 receptor positive allosteric modulators (PAMs) for the treatment of GABA_A_ α5 receptor-related diseases such as Alzheimer’s disease, mild cognitive impairment, bipolar disorders and many others [38]. Among isoxazole–isoxazole hybrids, the most active were two derivatives, compound **7a** and **7b** (Figure 2), that displayed Ki = 0.0085 µM and 0.039 μM, respectively, and thus strong binding affinity to the GABA α5/β3/γ2 receptor complex.

### 3.4. nAChR Modulators

The nicotinic acetylcholine receptor (nAChR) is a receptor classified as a ligand-gated cation channel that is involved in various functions of the central nervous system. For example, the neuronal nAChR provides fast synaptic transmission and releases neurotransmitters or cytokine in the brain and non-excitable cells. In particular, α7-containing nAChR modulates the survival and proliferation of non-excitable cells [39]. Crowley and colleagues prepared a library of spiropiperidines as allosteric modulators of nAChR [40]. All derivatives were evaluated for their inhibitory activity against α7 receptor type. In the study, compounds **8**, **9**, **10** and **11** (Figure 2) exhibited a half-maximal effective concentration (EC_50_) value equal to 0.016, 0.13, 0.12 and 1.4 µM, respectively. Due to its properties, these isoxazole–isoxazole and isoxazole–oxazole hybrids could find use for preventing, treating or ameliorating disease, particularly disorders of the central nervous system such as cognitive impairment in Alzheimer’s disease, Parkinson’s disease and schizophrenia.

### 3.5. SCD Inhibitors

Stearoyl-CoA desaturase (SCD) is an endoplasmatic reticulum enzyme involved in the biosynthesis of monounsaturated fatty acids (MUFAs) from saturated fatty acid (SFA). In humans, two isoforms of this enzyme are found, namely SCD1 and SCD5, which play a key role in lipid homeostasis, cell proliferation and survival. Moreover, both enzymes are crucial for neuronal differentiation, the regulation of synaptic function and myelination [41,42]. Wrona and coworkers designed isoxazole derivatives that can be used for treating or preventing neurological disorders such as Parkinson’s disease (PD) and Alzheimer’s disease (AD) [43]. Compounds were evaluated for their SCD1 and SCD5 inhibitory activity. It was determined that isoxazole–isoxazole hybrids **12** and **13** (Figure 2) showed a half maximal inhibitory concentration (IC_50_) value of 45 μM towards SCD1 and SCD5. In contrast, isoxazole–oxazole hybrid **14** (Figure 2) exhibited IC_50_ values of 19 and 10 µM against SCD1 and SCD5, respectively.

### 3.6. mGlu5 Receptor Modulator

Metabotropic glutamate receptor 5 (mGlu5 receptor) is a subtype in the group I mGlu receptors and, like the other mGlu receptor subtypes, is a member of the family 3/C G-protein-coupled receptors [44]. Burdi and coworkers obtained a series of novel compounds useful for the treatment, prevention and/or management of various disorders, such as neurological or psychiatric disorders, neuromuscular disorders, gastrointestinal disorders, lower urinary tract disorder and cancer [45]. In the mGluR5 in vitro functional assay, which was conducted using an aequorin cell line expressing human recombinant mGluR5 receptor, compound **15** (Figure 2) was found to modulate the receptor activity with IC_50_ > 10 µM.

## 4. Antibacterial Activity

**Figure 3:**
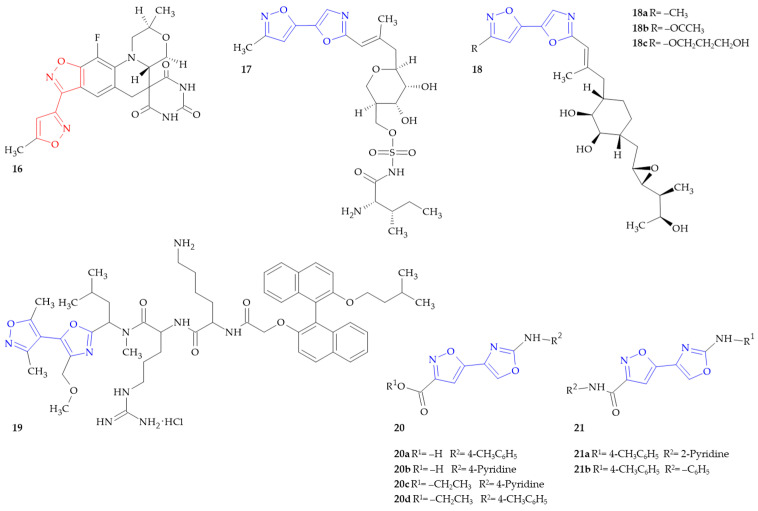
Isoxazole–(iso)oxazole hybrids with antibacterial activity.

The continuous development of new anti-infective therapeutics is required due to the accumulation of resistance in pathogenic organisms over time and with prolonged drug use. A bactericidal or bacteriostatic effect is the result of various mechanisms by which antibacterial agents act [46]. Such advancements may encompass novel compounds that are designed to target underexploited targets or modifications to existing drugs that are effective against exploited targets while simultaneously counteracting current resistance mechanisms. One group of enzyme targets that has been validated but remains underexploited is the class of aminoacyl-tRNA synthetases [47]. Understanding the mechanisms underlying both bacteriocidal and bacteriostatic effects allows us to design and synthetize effective antibacterial agents among isoxazole–(iso)oxazole hybrids (Figure 3).

The series of fused, spirocyclic heteroaromatic compounds were synthesized by Barvian (from AstraZeneca) et al. for the treatment of bacterial infections [48]. The in vitro IC_50_ of compound **16** (Figure 3) (dissolved in 0.8% DMSO) against *E. coli* DNA gyrase was 0.4 μM.

Best and colleagues prepared sulfamoyl-containing alditols as bactericides and t-RNA synthetase inhibitors [49]. Almost all compounds, including compound **17** (Figure 3), were generally found to be active against several representative strains of *S. pneumoniae*, with MICs in the range 1 to 32 µM/mL being obtained, as well as certain strains of *H. influenzae* (MICs 16–32 µM/mL) and *M. catarrhalis* (MICs 1–16 µM/mL).

Broom et al. synthesized semisynthetic analogs of pseudomonic acid A (Mupirocin) containing a heterocycle substituted oxazole [50]. Derivatives in which isoxazole–oxazole linked hybrids were present showed good antibacterial potency and were further evaluated in vivo. All of the obtained compounds were tested against different strains of bacteria, including *E. coli*, *S. aureus*, *S. pyogenes*, *S. pneumoniae*, *H. influenzae* and *M. catarrhalis*. Among isoxazole–oxazole derivatives, compounds **18a**, **18b** and **18c** (Figure 3) exhibited antibacterial activity against *E. coli* (MIC 128 μg/mL). The most active compound against *S. pyogenes* was compound **18a** (MIC 0.50 μg/mL), against *S. pneumoniae* it was compounds **18a** and **18b** (MIC 0.13 μg/mL), and against *H. influenzae*, the most active was compound **18c** (MIC 0.13 μg/mL).

Wales et al. investigated the antibacterial activity of a few series of compounds within the class of peptidomimetics [51]. In a recent study, the authors prepared a new binaphthyl-based, functionalized oxazole peptide. Among them, one derivative was an isoxsazole–oxazole hybrid **19** (Figure 3). Excellent antibacterial activity was observed for compound **19** across Gram-positive strains of bacteria, including *S. aureus*, MRSA, *E. faecalis* and *S. pneumoniae*, with minimal inhibitory concentrations (MICs) equal to 2, 2, 4 and 2 µg/mL, respectively. In comparison, vancomycin, which is an antibiotic used to treat Gram-positive bacterial infections, exhibited MICs of 1, 1, 4 and 0.5 µg/mL in this test. Compound **19** was also effective against Gram-negative strains, i.e., *E. coli*, with an MIC of 8 µg/mL, whereas the reference drug, i.e., chloramphenicol, showed an MIC of 4 µg/mL.

L-cysteine biosynthesis is a common way for bacteria and actinomycetales to develop a persistent infection and boost their resistance to antibacterial therapy. Subsequently, this could result in the emergence of antimicrobial resistance, which is currently one of the most alarming threats to public health on a global scale [52]. Since mammals lack the biosynthetic machinery needed to synthesize L-cysteine, using it as a target for medication shows great promise. Magalhães et al. reported a series of inhibitors of *Salmonella typhimurium* serine acetyltransferase (SAT), the enzyme that catalyzes the rate-limiting step of L-cysteine biosynthesis [53]. The discovery of substituted (2-aminooxazol-4-yl)isoxazole-3-carboxylic acids as inhibitors of bacterial serine acetyltransferase has been part of the quest for novel potential antibacterial adjuvants. Compound **20a** and **20b** (Figure 3) acted as a serine *O*-acetyltransferase inhibitor, with IC_50_ of 1.0 μM and 12.02 μM, respectively. Compound **20c** (Figure 3), the ethyl ester analog of compound **20b**, demonstrated activity with an IC_50_ value of 3.95 μM. Other isoxazole–oxazole linked derivatives also exhibited potent serine *O*-acetyltransferase inhibition activity, with IC_50_ in range of 1.54–8.08 μM.

Tuberculosis is one of the most lethal infectious diseases in the world, and the growing prevalence of multidrug-resistant and extensively drug-resistant strains is a cause for concern. It most often attacks the lungs, but can also affect other parts of the body [15]. Azzali et al. synthetized compound **20d** (Figure 3), with the structure of the aforementioned compound **20a** modified to an ethyl ester, which showed strong antibacterial activity against *Mycobacterium tuberculosis* (MIC 2.3 μM) [54]. Lilienkampf et al. have previously described the preliminary biological evaluation of a series of 2-aminothiazolyl isoxazoles with growth inhibitory activity against *Mycobacterium tuberculosis* strains [55]. Furthermore, these compounds were found to not be affected by the action of mycobacterial efflux pumps, unlike many molecules that are actually in clinical or preclinical evaluation [56]. The authors performed a detailed analysis of structural modifications that could provide excellent activity while maintaining the (thiazol-4-yl)isoxazole-3-carboxamide core, which is considered to be the pharmacophore of the molecule. Interestingly, Girardini and colleagues observed that the activity of compounds characterized by the 2-aminooxazole heterocycle instead of the 2-aminothiazole, was comparable to that of the parent compounds. The MIC_90_ values were 2.5 μg/mL and 1 μM for compound **21a** and **21b** (Figure 3), respectively [57].

## 5. Treatment of Cancer Diseases

**Figure 4:**
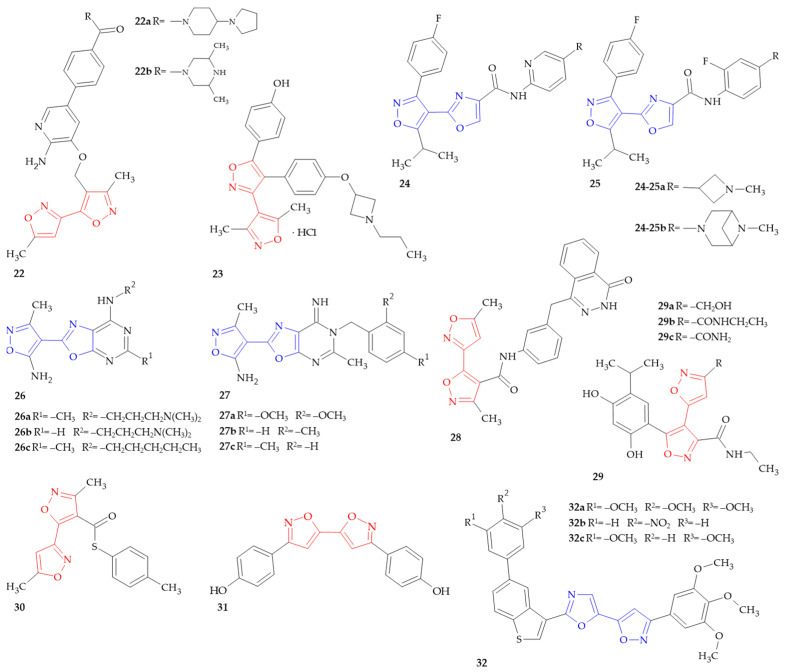
Isoxazole–(iso)oxazole hybrids with anticancer activity.

Cancer is a very significant global health problem. According to data provided by the World Health Organization (WHO), the numbers of new cancer cases and cancer deaths have increased each year [58]. Although many pharmaceuticals have already been approved to treat cancers, the development of resistance to existing drugs, serious adverse effects and unsatisfactory therapeutic efficacy highlight the need for new anticancer agents. The search for isoxazole–(iso)oxazole hybrids, which would act in a selective manner, is crucial in the context of cancer treatment (Figure 4). It should be noted that many of these compounds show anticancer effects through a targeted mechanism of action, including the inhibition of hepatocyte growth factor (HGF) receptor, TRAF2- and NCK-interacting kinase (TNIK), antiapoptotic proteins, vascular endothelial growth factor receptor-2 (VEGFR-2), poly (ADP-ribose) polymerase-1 (PARP1) and heat shock protein 90 (Hsp90). Furthermore, it was found that some of these hybrids act as estrogen receptor alpha (ERα) modulators and pregnane X receptor (PXR) antagonists. Finally, the mechanism of anticancer action has not been defined for a small number of compounds.

### 5.1. HGF Receptor Inhibitors

A broad class of distinct and powerful anticancer drugs known as kinase inhibitors targets protein kinases that are changed in cancer cells and partially responsible for the cells’ aberrant growth. These kinases are valuable tools in the treatment of certain cancers because they can disrupt the growth and spread of cancer cells by inhibiting them [59]. Cui and coworkers synthesized compounds of aminopyridine and aminopyrazine formula that exhibited protein kinase inhibitor activity [60]. In the group were also compounds containing isoxazole–isoxazole hybrids; biologically tested compounds **22a** and **22b** (Figure 4) inhibited hepatocyte growth factor (HGF) receptor at 25% and 22%, respectively.

### 5.2. ERα Receptor Modulators

Estrogen receptor alpha (ERα) belongs to the nuclear receptor superfamily of transcription factors that are primarily activated through binding to estrogen, specifically estradiol. ERα has been extensively studied in the context of hormone-dependent cancers, and most notably in breast cancer, where it is of significant prognostic and therapeutic value [61]. Dijcks and coworkers synthesized a series of *N*-substituted azetidine derivatives as a Selective Estrogen Receptor Modulator (SERM) [62]. As the authors state, the derivatives from the invention can be useful for the treatment of ovulatory dysfunction, uterine cancer, endometrium cancer, ovarian cancer, endometriosis, osteoporosis, prostate cancer, benign prostatic hypertrophy, breast cancer and hormone-treatment-resistant breast cancer. All compounds were evaluated for their ER modulating activity, and compound **23** (Figure 4) was shown as an agonist with an IC_50_ value of 0.0031623 µM. It should be emphasized that the mentioned derivatives with an *N*-substituted azetidine group have ERα antagonistic and—in certain embodiments—selective estrogen receptor downregulating (SERD) activity in ER-positive breast cancer cells.

### 5.3. TNIK Inhibitors

TRAF2- and NCK-interacting kinase (TNIK) is a serine/threonine kinase involved in various biological processes. In particular, it is an essential regulatory component of the Wnt signaling pathway, which plays important roles in carcinogenesis and embryonic development. The current data imply that TNIK inhibition is a potential target in colorectal cancer [63]. Zavoronkovs and coworkers designed series of various heterocyclic derivatives with potential TNIK inhibitory activity [64]. In the TNIK Human STE kinase enzymatic radiometric assay, isoxazole–oxazole hybrids **24a**, **24b**, **25a** and **25b** (Figure 4) showed IC_50_ values ranging within 12–150 nM. The promising results make it possible to use these compounds in cellular proliferation-related diseases.

### 5.4. Antiapoptotic Protein Inhibitors

B-cell lymphoma 2 (BCL-2) is an anti-apoptotic protein which regulates cytochrome c release at the mitochondria. BCL-2 inhibition resulted in the loss of the anti-apoptotic defense mechanism of cancerous cells, leading to their apoptosis, i.e., a process of programmed cell death [65]. The two series of novel isoxazole-containing hybrids were synthesized by Sochacka-Ćwikla et al. as anticancer agents [66,67]. The obtained compounds were evaluated for their cytotoxic activity against a panel of human cancer cell lines (A-431 epidermal, A549 lung, MCF7 breast, HT29 primary colon, LoVo metastatic colon) and one mouse cancer cell line (L1210 leukemia). Activity comparable to the reference drug, i.e., cisplatin, was found for derivatives **26a** and **26b** (Figure 4) from series I and II, respectively. Additionally, derivative **26b** was less toxic to healthy human dermal fibroblasts (NHDF) than cisplatin. Compound **26c** (Figure 4) suppressed the synthesis of anti-apoptotic protein BCL-2 and compound **26b** decreased the BCL-2 level in the HT29 cell line and inhibited the migration of HT29 cells. Furthermore, compound **26a** increased the expression of components of mitogen-activated protein kinase pathways (MAPKs) such as JNK, p38α and p38β. To conclude, the tested compounds were associated with the initiation of cell signaling pathways leading to cell apoptosis.

### 5.5. VEGFR2 Inhibitors

**Figure 5:**
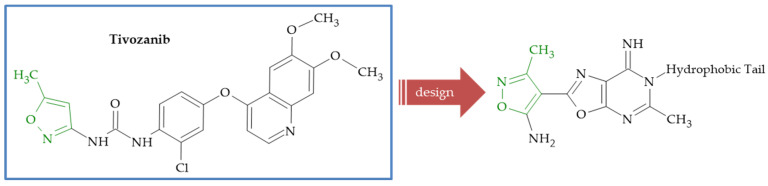
Design of isoxazole-substituted 6-*N*-benzylooxazolo[5,4-*d*]pyrimidin-7(6*H*)-imines based on tivozanib.

Vascular endothelial growth factor receptor-2 (VEGFR-2) is tyrosine kinase receptor (RTK) involved in tumor angiogenesis via the modulation of vascular permeability, endothelial cell migration, proliferation and survival. The kinase is frequently mutated and/or overexpressed in various types of cancers, namely lung, breast and colorectal carcinoma [68]. The series of 6-*N*-benzyloxazolo[5,4-*d*]pyrimidin-7(6*H*)-imines **27** with potential VEGFR2 inhibitory activity was synthetized by Sochacka-Ćwikła et al. [69]. The obtained derivatives were evaluated in vitro for cytotoxic activity against a normal cell line, i.e., normal human dermal fibroblasts (NHDF), and four cancer cell lines characterized by the overexpression of the VEGFR2 receptor (lung A549, colon HT29, melanoma A375 and breast MCF7). The study results indicated that compound **27a** (Figure 4) showed activity comparable to the reference drug, i.e., tivozanib (Figure 5), against all tested cancer lines, and compounds **27b** and **27c** (Figure 4) showed activity comparable against three of them (Table 1). It is worth noting that derivative **27c** showed no cytotoxicity against the healthy cell line NHDF. Compounds **27a** and **27c** proved to be effective VEGFR2 inhibitors, with IC_50_ values comparable to tivozanib. Moreover, compounds **27a** and **27b** significantly inhibited the tube-forming ability of human microvascular endothelial cells of the skin (HMEC-1), indicating their anti-angiogenic potential.

### 5.6. PARP1 Inhibitors

The efficacy of cancer treatment can be increased using molecules that have specific cellular targets, such as Poly (ADP-ribose) polymerase-1 (PARP1), which are involved in DNA repair. PARP is a protein that can be found in cells and whose role is to help damaged cells to repair themselves. Among PARP families, PARP1 exhibits abundant expression in comparison to the others and it seems to be responsible for most of the cellular PAR formation. Therefore, most studies have focused on PARP1 [70]. Hawkins and coworkers discovered that certain derivatives of 1(2*H*)-phthalazinone and related compounds exhibited an inhibition of PARP activity. Compound **28** (Figure 4) inhibited PARP IC_50_ ≤ 1 μM [71].

### 5.7. Hsp90 Inhibitors

**Figure 6:**
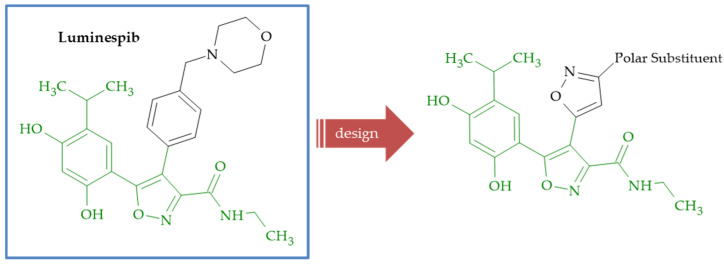
Design of isoxazole–isoxazole hybrids targeting Hsp90 based on luminespib.

An ATP-dependent molecular chaperone, heat shock protein 90 (Hsp90), regulates the stability and activity of client proteins that play pivotal roles in tumor proliferation, survival and transformation, demonstrating that Hsp90 inhibition is a promising strategy for anticancer therapy. In order to find new anticancer agents, a series of isosteric surrogates of the 4-phenyl group in luminespib (Figure 6) were investigated as new scaffolds of the Hsp90 inhibitor [72]. Among the synthesized surrogates of isoxazole, compounds **29a** and **29b** (Figure 4) exhibited potent Hsp90 inhibition in ATPase activity and Her2 degradation assays and also significant anticancer activity in the A2780 and HCT116 cell lines. Animal studies indicated that compound **29a** and **29b** demonstrated better or comparable activity than luminespib in A2780 or NCI-H1975 tumor xenograft models. A molecular modeling study demonstrated that compound **29a** could fit nicely into the *N*-terminal ATP binding pocket. Kang and coworkers previously obtained and described anticancer activity of novel five-membered heterocycle derivatives as Hsp90 inhibitors [73]. Among all of the synthesized compounds, 41 were isoxazole–isoxazole derivatives, and the most active were compounds **29a** and **29c** (Figure 4). In summary, the biological data of isoxazole–isoxazole hybrids **29a**, **29b** and **29c** is shown in Table 2.

### 5.8. PXR Antagonist

Pregnane X receptor (PXR) is a member of the NRII nuclear receptor family and acts as a xenobiotic sensor and a paramount transcriptional regulator of drug-metabolizing enzymes and transporters. Its overexpression in various types of cancers indicates the importance of PXR as a therapeutic target for countering multidrug resistance in anticancer treatments [74]. Hodnik et al. presented the discovery of novel bazedoxifene-scaffold-based PXR antagonists inspired by the marine sulfated steroids solomonsterol A and B as natural leads [75]. Among all compounds, derivative **30** (Figure 4) stands out as the most potent PXR antagonist so far, with an IC_50_ value of 850 nM in PXR-transfected HepG2 cells.

### 5.9. Undefined Mechanism of Action

Gothelf presented an invention related to bis-heterocyclic derivatives having anticancer properties [11]. The NCI in vitro primary anticancer screening of compound **31** (Figure 4), bis-5,5′-(3-(4′′-hydroxyphenyl)-isoxazole), against 60 cell lines was performed. The tests were conducted at a minimum of five concentrations at 10-fold dilution and a sulforhodamine B (SRB) protein assay was used to estimate cell viability or growth. The determined response parameters were growth inhibition 50% (GI_50_), total growth inhibition (TGI), and lethal concentration 50% (LC_50_). Compound **31** exhibited GI_50_ of less than 1 µM for CCRF-CEM leukemia, HTC-15 colon cancer and SN12C renal cancer cell lines. In the case of TGI, the best results were observed for MOLT-4 leukemia and SF-539 CNS cancer cell lines, with values of 22.0 and 24.7 µM, respectively. It is worth noting that compound **31** was found to be non-toxic in the study.

The series of 3-(3,4,5-trimethoxyphenyl)-5-(2-(5-arylbenzo[b]thiophen-3-yl)oxazol-5-yl)isoxazole derivatives as anticancer agents was designed and synthetized by Premalatha and colleagues [76]. The obtained compounds were evaluated for their cytotoxic activity against four human cancer cell lines, i.e., breast MCF-7 and MDA MB-231, lung A549 and prostate DU-145, by using an MTT assay. Most of the compounds, especially derivative **32a**, **32b** and **32c** (Figure 4), exhibited good anticancer activity (Table 3). Additionally, half of them were more potent than the reference drug, i.e., etoposide. Interestingly, compound **32b** with the 4-nitro substituent displayed excellent anticancer activity towards all tested cancer lines.

## 6. Anti-Inflammatory Activity

**Figure 7:**
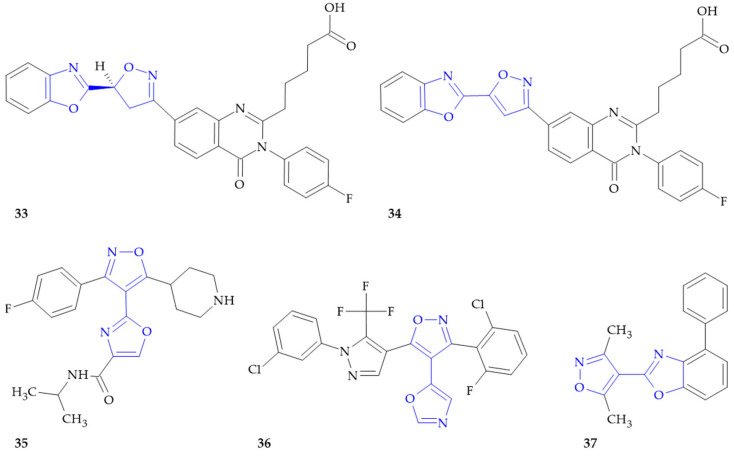
Isoxazole–oxazole hybrids with anti-inflammatory activity.

The development of novel isoxazole–oxazole hybrids with anti-inflammatory activity plays a key role in treating common inflammatory diseases, including chronic respiratory diseases, autoimmune disease, neurodegenerative disorders and cardiovascular conditions (Figure 7). The therapeutic potential of these compounds is a result of their ability to target several inflammatory pathways, such as the inhibition of the chemoattractant receptor-homologous molecule expressed on Th2 cells (CRTh2) receptor, p38 kinase, alpha-protein kinase 1 (ALPK1) and the production of interleukin 17 (IL-17) and interferon gamma (IFN-γ).

### 6.1. CRTh2 Antagonists

The constant need to search for new medicines in the treatment of chronic respiratory diseases such as asthma, COPD and allergic rhinitis has prompted the research and development of CRTh2 receptor antagonists as novel therapy [77]. One of the two types of G-protein-coupled receptors (GPCRs) that mediates the biological effects of prostaglandin D2 (PGD2) is the chemoattractant receptor-homologous molecule expressed on Th2 cells (CRTh2) receptor. PGD2 is a hormone-like metabolite in the arachidonic acid pathway that is known to be involved in allergic inflammation. PGD2-induced chemotaxis, degranulation and cytokine production have been shown to be mediated exclusively via CRTh2 receptors. Xiao et al. presented the synthesis and pharmacological and physicochemical characterizations of isoxazoline amide mimics as potent, selective CRTh2 antagonists with great pharmacokinetic profiles [78]. Compound **33** (Figure 7) has been shown to be an antagonist of prostaglandin D2 receptor 2 (Ki ≤ 0.01 µM). Interestingly, only the S-enantiomer was biologically active. Another group of researchers obtained quinazolinone derivatives that also acted as CRTh2 antagonists, including compound **34** (Figure 7) with K_i_ ≤ 0.01 µM [79].

### 6.2. p38 Kinase Inhibitors

p38 kinases are serine/threonine kinases of the MAPK (mitogen-activated protein kinase) signaling pathway, and play a crucial role in cell proliferation, cell death and regulation of the production of inflammatory cytokines. These kinases, especially p38α, are activated in response to extracellular stress signals. The inhibition of p38 kinases is provided for the treatment or prevention of diseases such as inflammatory diseases, autoimmune diseases, cancer, cardiovascular disease, infectious diseases, neurodegenerative diseases and metabolic diseases [80]. Severance and colleagues synthetized a library of ortho-terphenyl heterocyclic derivatives as inhibitors of p38 kinase [81]. Of nearly 2200 compounds from the invention, isoxazole–oxazole hybrid **35** (Figure 7) exhibited p38α kinase IC_50_ values of less than 1 μM. Overall, the results of the p38 inhibition assay revealed that the compounds from the invention generally exhibited IC_50_ values of around 30 μM and below. This finding suggests that these compounds could be considered as potent p38 inhibitors.

### 6.3. IL-17 and IFN-γ Production Inhibitors

Interleukin 17 (IL-17) and interferon gamma (IFN-γ) are pro-inflammatory cytokines inducing and modulating immune responses. IL-17 is responsible for T cell and neutrophil activation upon bacterial or fungal infection, since IFN-γ promotes macrophage activation and controls cellular proliferation and apoptosis. The activity of these cytokines is also associated with the development of autoimmune diseases [82,83]. IL-17 and IFN-γ production inhibitors were identified among a series of pyrazolylisoxazole derivatives by Leban and colleagues [84,85]. An analysis of proliferation of human peripheral blood mononuclear cells (PBMC) stimulated with phytohemagglutinin (PHA) and cytokine production, including IL-17 and IFN-γ, was conducted. Among the tested compounds, isoxazole–oxazole hybrids exhibited good biological activity, with the most promising results observed for compound **36** (Figure 7). This derivative demonstrated an inhibition of T cell proliferation as well as IL-17FF and IFN-γ production with IC_50_ ≤ 0.01 µM. The compound may be useful in the treatment of various inflammatory and autoimmune disorders, for example psoriasis, multiple sclerosis, rheumatoid arthritis or Crohn’s disease.

### 6.4. ALPK1 Inhibitors

Alpha-protein kinase 1 (ALPK1), a member of a subfamily of the atypical protein kinase family, is a key regulator in inflammation. During bacterial infection, ALPK1 is activated by bacterial nucleoside diphosphate heptoses, resulting in the initiation of downstream signaling of the innate immune response. In contrast, diseases displaying mutations in ALPK1, such as ROSAH syndrome or spiradenoma, lead to chronic inflammatory signaling without infection [86]. Du and colleagues prepared a library of heterocyclic derivatives as inhibitors of alpha-kinase 1 (ALPK1) [87]. An investigation of both the ALPK1 kinase activity in the 293T cells and the ALPK1 kinase activity in the in vitro kinase assay was performed. The isoxazole–oxazole hybrid **37** (Figure 7) inhibited FL (full-length)-ALPK1 activity in the NF-κB luciferase reporter assay, with an IC_50_ value higher than 200 µM, and ALPK1 activity in the culture media with an IC_50_ value higher than 100 µM. These findings make it possible to state that the tested derivatives have the potential for use in the management of a wide range of an inflammation-related diseases, including ROSAH syndrome, inflammatory bowel disease, non-alcoholic steatohepatitis, gout, diabetes, chronic kidney disease, pancreatitis, Kawasaki disease or inflammatory skin diseases. The authors also state that the compounds can be useful for treating neurodegenerative diseases such as Alzheimer’s disease.

## 7. Treatment of Metabolic Diseases

**Figure 8:**
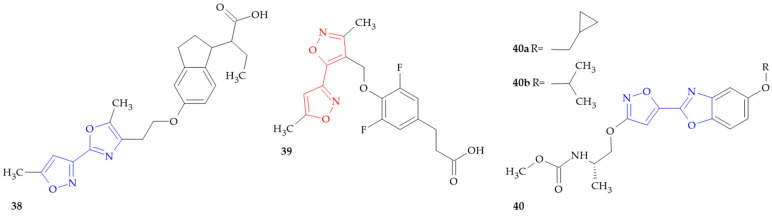
Isoxazole–(iso)oxazole hybrids to treat metabolic disorders.

In the 21st century, metabolic diseases such as type 2 diabetes, obesity, hypertension and hypercholesterolemia are increasingly being recognized as a global health challenge, due to their strong association with modern lifestyle, characterized by inactivity and poor diet. These disorders require the regulation of multiple metabolic pathways that are directly related to lipid and glucose metabolism and/or insulin levels, indicating the constant need for effective, multitargeted agents [88]. Isoxazole–(iso)oxazole hybrids are a promising class of compounds that could overcome this therapeutic challenge (Figure 8). The resulting derivatives were found to activate peroxisome proliferator-activated receptors (PPARs) or G-protein-coupled receptor 120 (GPR120) and inhibit acetyl-CoA carboxylase (ACC).

### 7.1. PPARs Agonists

Peroxisome proliferator-activated receptors (PPARs) are molecular targets of great importance for glucose and lipid metabolism, insulin sensitivity or immune response [89]. The series of oxazole-substituted indanylacetic acids evaluated as PPARs agonists were designed by Lowe and coworkers [90]. The study was first conducted with fluorescence resonance energy transfer (FRET) assays, using human ligand-binding domains for PPAR-α and PPAR-γ, and the co-activator CREB-binding protein. The best compounds were then profiled in a cellular transactivation assay. Isoxazole–oxazole hybrid **38** (Figure 8) was present in the study, with EC_50_ values of 827 and 762 nM against PPAR-α and PPAR-γ, respectively. The study results highlight the importance of the development of dual-acting PPAR-α-γ ligands for the treatment of metabolic diseases related to impaired glucose and lipid homeostasis.

### 7.2. GPR120 Receptor Agonists

Diabetes mellitus has become a serious health problem worldwide because it is connected to multiple complications such as cardiovascular diseases, nerve and/or kidney damage and eye damage (retinopathy or even blindness) [91]. There are two main types of diabetes mellitus: 1 (insulin-dependent) and 2 (non-insulin-dependent). G-protein-coupled receptors (GPRs) are important signaling molecules that are involved in many functions. Several studies have shown that GPRs such as GPR120 receptor play an important role in regulating blood glucose homeostasis. The free fatty acid receptor 4 (GPR120 or FFAR4) is a seven-transmembrane GPCR that is mainly expressed in the intestine and other tissues, such as adipose tissue, and macrophages [92]. GPR120, which is activated by free fatty acids (such as omega-3 fatty acids), can stimulate the release of glucagon-like peptide-1 (GLP-1) which is a potent incretin hormone that increases the glucose-dependent insulin secretion of insulin from pancreatic beta cells. GPR has become a promising target for the treatment of obesity, diabetes mellitus type 2 and metabolic syndrome, considering the significant role of GLP-1 in insulin secretion, gastric emptying and appetite feeding control. [(Heterocyclylmethoxy)aryl]alkanoic acid derivatives have been described as GPR120 agonists [93]. Compound **39** (Figure 8) was found to be useful for the treatment of metabolic diseases, including Type II diabetes and diseases associated with poor glycemic control presenting EC_50_ ≤ 1 μM.

### 7.3. Acetyl-CoA Carboxylase Inhibitors

Acetyl-CoA carboxylase (ACC) is an allosteric enzyme that catalyzes the primary regulating step in the synthesis of fatty acids [94]. Compounds with an ACC inhibitory action are useful for the prophylaxis or treatment of metabolic syndrome, obesity, hypertension, diabetes, cardiovascular diseases associated with atherosclerosis and the like. Yasuma and coworkers presented bicyclic compounds, and among all those with isoxazole–oxazole derivatives, it inhibited Acetyl-CoA carboxylase 2, compounds **40a** and **40b** (Figure 8) (92% and 85%, respectively) [95].

## 8. Other Biological Activity

**Figure 9:**
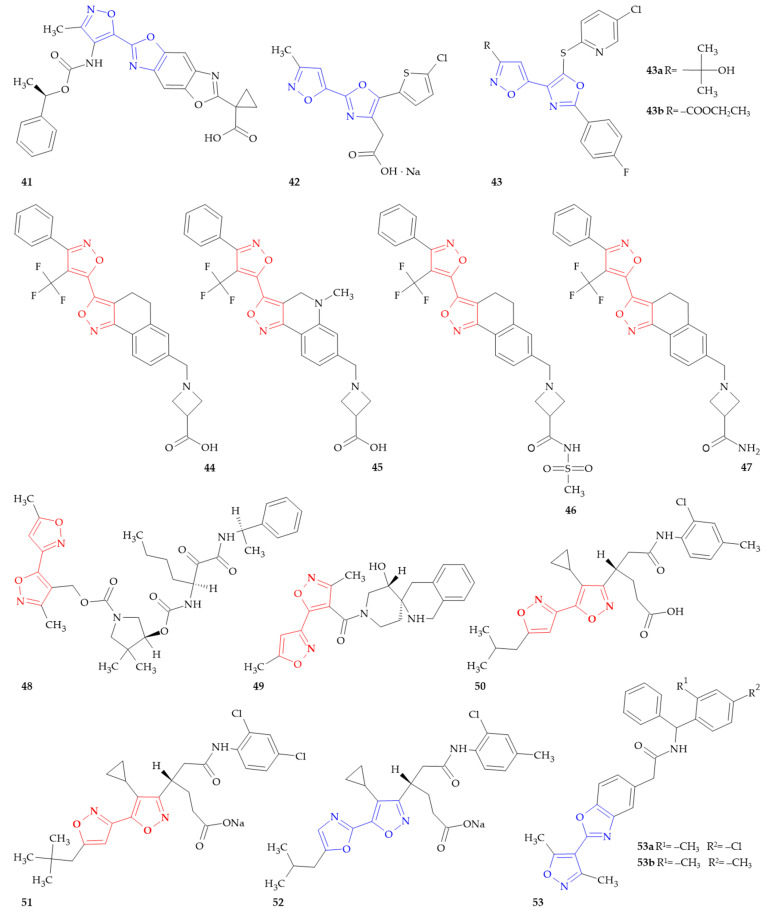
Isoxazole–(iso)oxazole hybrids with various biological activities.

Due to their structural diversity, isoxazole–(iso)oxazole hybrids exhibit a broad range of biological activities (Figure 9). These compounds are known to act as lysophosphatidic acid (LPA) receptor antagonists and calcium-activated K channel openers, fatty acid amide hydrolase (FAAH) inhibitors, sphingosine 1-phosphate 1 (S1P1) agonists, cathepsin K inhibitors, protein arginine methyltransferase 5 (PRMT5) inhibitors and retinoic acid-related orphan receptor gamma (RORγ) modulators. This finding lends further support to their therapeutic value.

### 8.1. LPA Receptor Antagonists

Lysophosphatidic acid (LPA) is a lysophospholipid that has been demonstrated to act through sets of specific G-protein-coupled receptors (GPCRs) in an autocrine and paracrine fashion [96]. LPA acts as a messenger both inside and outside cells and regulates various cellular responses such as proliferation, apoptosis, migration and secretion. LPA binds to its cognate GPCRs (LPA_1_-LPA_6_) and activates intracellular signaling pathways to produce a variety of biological responses, and in that way antagonists of the LPA receptors can be employed in the treatment of diseases or disorders in which LPA plays a role. Buckman’s research team [97] synthesized a series of *N*-heterocyclylcarbamates that acted as lysophosphatidic acid receptor 1 antagonists. Compound **41** (Figure 9), in vitro, had EC_50_ of 0.05–0.5 µM.

### 8.2. Calcium-Activated K Channel Openers

Ca^2+^-activated K^+^ (K_Ca_) channels are a distinctive family of ion channels that are capable of directly communicating calcium signals for changes in cell membrane potential that are necessary for cellular processes, such as cellular proliferation and migration [98]. Almost all vertebrate cells contain potassium channels, and the potassium influx through these channels is essential for the preservation of a hyperpolarized resting membrane potential. Hongu et al. presented a series of compounds that acted as an excellent large conductance calcium-activated K channel opener containing a nitrogen-containing five-membered heterocyclic [99]. The EC_50_ of compound **42** (Figure 9) required for the relaxation of potassium-induced contractions in the presence of 0.1 µM papaverine in isolated rabbit urinary bladder is ≤1 µM.

### 8.3. FAAH Inhibitors

Fatty acid amide hydrolase (FAAH) is an enzyme that breaks down endocannabinoids like anandamide (AEA) and may provide beneficial effects in a mouse model of Alzheimer’s disease (AD)-like pathology [100]. Studies suggest that FAAH plays a role in a variety of diseases, including gastrointestinal disorders, pain, mood disorders and even some types of cancer [101]. Chobanian et al. prepared pyridinylsulfanyloxazole derivatives and analogs for use as FAAH inhibitors [102]. Compounds **43a** and **43b** (Figure 9), obtained by the authors, acted as inhibitors of fatty-acid amide hydrolase 1 (IC_50_ = 0.035 μM and 0.02 μM, respectively), and in this aspect these derivatives can be useful in the treatment of pain and other FAAH-mediated diseases, and disorders like osteoarthritis, rheumatoid arthritis, diabetic neuropathy, postherpetic neuralgia, fibromyalgia, migraine, sleep disorder, Alzheimer’s disease and Parkinson’s disease.

### 8.4. S1P_1_ Agonists

The sphingosine 1-phosphate (S1P) signaling pathways possess important and diverse functions. Consequently, S1P receptors (S1PRs) have been identified as a therapeutic target for a range of diseases, because of their ability to regulate lymphocyte trafficking, brain and cardiac function, vascular permeability, and vascular and bronchial tone [103]. The initial development of S1PR modulators was to prevent immune system rejection following renal transplantation, but the current approved indication is for multiple sclerosis. There are four S1PR modulators with regulatory approval for multiple sclerosis (fingolimod, siponimod, ozanimod and ponesimod). Evidence from preclinical studies and ongoing and completed clinical trials supports the development of S1PR modulators for other therapeutic indications. The efficacy of the nonselective S1P receptor agonist fingolimod, which was approved by the FDA in 2010 for the treatment of relapsing remitting multiple sclerosis (RRMS), has sparked interest in its clinical evaluation and the addition of additional S1P_1_ agonists for a growing list of disorders (Crohn’s disease, ulcerative colitis, psoriasis, polymyositis, progressive multiple sclerosis, lupus) [104]. Murali Dhar and coworkers patented a series of tricyclic heterocyclic compounds useful as S1P_1_ agonists [105]. Among them there were 12 hybrids of isoxazole–isoxazoles and one of isoxazole–oxazole. The most active compound was compound **44** (Figure 9) (with EC_50_ against S1P_1_ and S1P_3_ equal to 3.2 × 10^−5^ µM and >31 µM, respectively), which also acted as an inhibitor of cytochrome P450 (14–40 μM). A structurally similar derivative **45** (Figure 9) showed potent activity against S1P_1_ (with EC_50_ value of 0.0016 μM), but weaker activity against S1P_3_ (with EC_50_ > 63 µM). For example, one of the most active derivatives was also compound **46** (Figure 9), which acted as an agonist of S1P_1_ and S1P_3_ with the values EC_50_ ≤ 0.015 μM and ≥3.5 μM, respectively. These compounds may by used in treating, preventing or slowing the progression of diseases in a variety of therapeutic areas, such as autoimmune diseases and vascular diseases.

**Figure 10:**
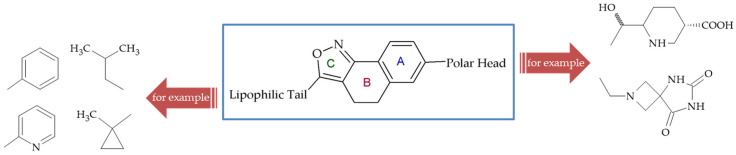
Tricyclic agonists of aphingosine-1-phosphate receptor 1 (S1P_1_) employing ligand-based drug design (A, B and C indicate phenyl ring, central ring and heterocyclic ring, respectively).

Xiao and coworkers [106] continued searching for S1P_1_ agonists and identified a series of potent tricyclic agonists of S1P_1_ with selectivity over S1P_3_ which were efficacious in a pharmacodynamics model of suppression of circulating lymphocytes. The authors thoroughly discussed the structure–activity relationship (SAR), taking into account different substituents in the novel scaffold of the polar head piece, phenyl ring (A), central ring (B), heterocyclic ring (C), and the lipophilic tail piece (Figure 10). The results indicated that compound **47** (Figure 9), an isoxazole–isoxazole hybrid with an azetidine carboxylic acid as the polar head moiety, exhibited the desired pharmacokinetic and pharmacodynamic profile. Furthermore, it displayed maximal efficacy when administered orally in a rat adjuvant arthritis model, with an ED_50_ value of 24 nM, which is comparable to reported S1P_1_ modulators. Finally, this compound was identified as exhibiting the optimal properties in terms of potency for S1P_1_ (EC_50_ = 0.59 ± 0.19 nM), selectivity in comparison to S1P_3_ (EC_50_ = 4400 ± 330 nM), and an excellent profile in liability assays (metabolic stability, CYP inhibition, etc.). The authors proved that of the various heterocyclic rings examined, the [1,2-*c*]isoxazole is the most potent in the S1P_1_ GTPγS assay. They showed also an application of a ligand-based drug design approach to the discovery of potent agonists of S1P_1_ with selectivity over S1P_3_, which were efficacious in a pharmacodynamic model of suppression of circulating lymphocytes.

### 8.5. Cathepsin K Inhibitors

Cathepsin plays a crucial role in bone resorption and has emerged as a prominent therapeutic target for the treatment of bone-related diseases such as osteoporosis [107]. Ketoamides were found to be used as inhibitors of cathepsin enzymes, particularly cathepsin K and cathepsin S. They have been developed as potential therapeutics for diseases like osteoporosis and cancer, where these enzymes play a role. Barrett et al. focused on optimizing ketoamide structures to improve their potency and synthesized a series of 1-(oxoaminoacetyl)pentylcarbamate derivatives [108]. Compound **48** (Figure 9) showed a potency to inhibit cathepsin K, with a value IC_50_ 0.1 μM.

### 8.6. PRMT5 Inhibitors

Protein arginine methyltransferase 5 (PRMT5) acts by catalyzing the symmetric demethylation of arginine residues on different proteins, including histone and non-histone proteins. It has been demonstrated that PRMT5 plays an important role in epigenetic regulation, DNA repair and RNA processing. A high expression of PRMT5 was observed in human cancers [109]. Machacek and coworkers designed and synthesized a series of potent PRMT5 inhibitors. In the assay, cell potency expressed as the EC_50_ value of each compound was determined and it was shown that compound **49** (Figure 9) demonstrated an EC_50_ (inhibiting PRMT5) value of 0.297 μM [110].

### 8.7. RORγ Modulators

Recent studies showed that retinoic acid-related orphan receptors (RORs), RORγ among others, demonstrate a wide binding specificity for a number of sterols [111]. Some cholesterol intermediates or metabolites can act as ligands of ROR, and in that way the changes in cholesterol homeostasis have an impact on the transcriptional activity of ROR and finally can result in changes in the processes regulated by RORs, like immune responses, inflammation, cancer or several neurological disorders. Extensive research was conducted to explain the relationship between cholesterol metabolism, RORγ activity and their regulation of Th17 differentiation and autoimmune disease. The studies suggest that RORγt (the shorter isoform of RORγ) may be a promising therapeutic target for the treatment of a variety of inflammatory diseases [112]. Almost 10 years ago, Kotoku and coworkers synthesized isoxazole derivatives that acted as RORγ antagonists. They found that compound **50** (Figure 9) can act as an inhibitor of nuclear receptor RORγ (EC_50_ values ranging from 0.0014 μM to 3 μM) and in that way it can be used in the treatment of different autoimmune diseases, like Crohn’s disease or rheumatoid arthritis [113].

Kotoku et al. [114] continued their search for RORγ inhibitors made up of an azole scaffold. They examined the influence of different motifs, and finally they discovered the second generation RORγ inhibitor made up of a 4-(isoxazol-3-yl)butanoic acid scaffold, i.e., compound **51** (Figure 9). The calculated effective dose 50% (ED_50_) value for compound **51** in the pharmacodynamic model was 3 mg/kg, achieving roughly a 10-fold improvement in comparison to the earlier-reported RORγ inhibitor, whose structure was based on the azole scaffold but did not contain the oxazole/isoxazole moiety. To further confirm the efficacy of this compound, disease-modifying effects were analyzed in the IL-23-induced mouse dermatitis model. A high RORγ specificity of compound **51** was also confirmed by the absence of inhibitory activity against other nuclear receptors (EC_50_ > 20 μM; hRORα, mLXR, hRXR, hPPARδ, hPPARγ) and the lack of time-dependent CYP inhibition properties (IC_50_ > 50 μM; hCYP3A4m, hCYP3A4t, hCYP2C9, hCYP2D6, hCYP1A2, hCYP2C19). Compound **51** showed greatly improved pharmacokinetic profiles, suggesting it was indeed a promising preclinical candidate as a selective RORγ inhibitor. Among all of the obtained compounds, isoxazole–oxazole linked derivative, compound **52** (Figure 9) exhibited also strong inhibitory properties (EC_50_ = 0.66 μM).

The Baloglu research group was the first to discover RORγ modulators in the class of isoxazole–oxazole hybrids [115]. The RORγ modulating activity was evaluated using a dual fluorescence energy transfer (FRET) assay. All tested derivatives, for example compound **53a** and **53b** (Figure 9), were found to have an IC_50_ between 0.001 and 15.8 µM. They can therefore be considered for the treatment of diseases mediated by RORγ, such as multiple sclerosis, rheumatoid arthritis, acute myeloid leukemia, melanoma or psoriasis.

## 9. Conclusions

This review summarizes the advances in molecular hybridization of isoxazole and oxazole rings reported from 1995 to the end of 2024, i.e., over a period of thirty years. The analysis of the literature presented herein confirms the status of isoxazole–(iso)oxazole hybrids as exceptionally versatile privileged scaffolds in medicinal chemistry. The presence of diverse biological activities, for example anticancer, antibacterial, antitubercular, anti-inflammatory and antidepressive effects, across numerous studies, substantiates the hypothesis that molecular hybridization of these *N*,*O*-heterocyclic units amplifies or synergizes pharmacological profiles relative to the parent monomers. This finding is further confirmed by the fact that these hybrids inhibit various enzymes and receptors, including the P2Y_1_ receptor, GATA4, OX2R, SCD, ACC, ALPK1, FAAH, PRMT5, LPA, PXR, CRTh2 receptor, p38 kinase and cathepsin K. Furthermore, these compounds modulate the activity of many other receptors, such as RORγ, GABA_A_α5, Erα, GPR120, PPARs, nAChR and mGlu5.

The biological activity of isoxazole–(iso)oxazole hybrids appears to be influenced by the relative position of linkages between the heterocyclic rings. In particular, compounds in which the isoxazole ring is substituted at position 3 and connected to an isoxazole ring at position 5 were among the most frequently studied hybrids. This specific connection may favor a molecular conformation that enhances interaction with biological targets, such as enzymes or receptors, and promotes the occurring of biological activity. Moreover, isoxazole–isoxazole hybrids connected via alternative positions, i.e., 5-5, and isoxazole–oxazole hybrids with 4-2 or 5-2 linkages have been the subject of many biological tests. It should be emphasized that, among the described hybrids, only the isoxazole–oxazole connection showed anti-inflammatory activity. In contrast, only isoxazole–isoxazole hybrids were found to be agents for treating blood and heart diseases. Interestingly, no biological studies have been carried out on isoxazole–(iso)oxazole hybrids connected via positions 4-4, as well as isoxazole–oxazole hybrids with 3-4 and 3-5 linkages. Furthermore, hybrid compounds formed after ring linkage via heteroatoms have not been obtained at all. This may be due to two factors; firstly, the low probability of biological activity, and secondly, the relative difficulty of their synthesis or the limited opportunities for functionalization. These preliminary observations suggest that the regiochemistry of ring coupling plays a significant role in biological response modulation.

Isoxazole–(iso)oxazole hybrids, by integrating two or more pharmacophoric moieties into a single compound, offer the possibility of improving selectivity, enhancing potency and potentially reducing side effects. Importantly, the subtle modifications concerning linker flexibility, substituent electronics and regioisomerism may enhance target specificity. However, the development of hybrid compounds is associated with several significant challenges. Potential limitations may include the complication of the design and synthesis process, the pharmacokinetic profile of compounds (e.g., poor solubility) and the costs of their production [116]. In certain cases, hybrid molecules may exceed the criteria defined by Lipinski’s Rule of Five, particularly in terms of molecular weight and lipophilicity. This can result in a reduction in their oral bioavailability and ability to cross cell membrane bilayers. The stability of hybrids is a further challenge, the extent of which is dependent on the selected linker, especially cleavage linkers. The role of the linkers is essential in drug delivery systems, as they provide compound stability in systemic circulation and release efficiency in the target tissue. Despite its limitations, molecular hybridization represents a powerful tool for the rational design of multitarget agents with improved therapeutic potential.

## Figures and Tables

**Table 1:** In vitro activity of 6-*N*-benzyloxazolo[5,4-*d*]pyrimidin-7(6*H*)-imines as VEGFR2 inhibitors. Data adapted from Sochacka-Ćwikła [69].

	IC_50_ ± SD * [µM]					
	A549	HT-29	A375	MCF7	NHDF	VEGFR2
Tivozanib	10.03 ± 4.25	8.38 ± 4.04	**7.28 ± 3.35**	7.44 ± 3.51	7.10 ± 3.74	15.7 ± 5.9
**27a**	20.91 ± 7.60	19.70 ± 3.55	9.63 ± 2.41	17.43 ± 4.63	20.71 ± 6.92	3.73 ± 2.8
**27b**	23.58 ± 8.67	**38.36 ± 5.98**	40.16 ± 9.21	5.33 ± 2.45	27.85 ± 10.92	51.07 ± 10.1
**27c**	61.02 ± 17.04	149.06 ± 60.33	**111.75 ± 21.21**	6.12 ± 2.87	>100	18.65 ± 6.8

* The values are expressed as mean ± standard deviation (SD) with statistical significance compared to tivozanib marked in bold (*p* < 0.05).

**Table 2:** In vitro activity of the isoxazole–isoxazole hybrids as Hsp90 inhibitors. Data adapted from Kang [73].

	IC_50_ [µM]			
	ATPase	Her2	A2780	HCT116
Luminespib	0.500	0.012	0.006	0.02
**29a**	0.279	0.042	0.005	0.013
**29b**	0.284	0.028	0.043	0.014
**29c**	0.657	0.052	0.015	0.079

**Table 3:** In vitro cytotoxicity of 3-(3,4,5-trimethoxyphenyl)-5-(2-(5-arylbenzo[b]thiophen-3-yl)oxa-zol-5-yl)isoxazole derivatives. Data adapted from Premalatha [76].

	IC_50_ [µM]			
	MCF-7	A549	DU-145	MDA MB-231
Etoposide	2.11 ± 0.024	3.08 ± 0.135	1.97 ± 0.45	1.91 ± 0.84
**32a**	0.33 ± 0.08	0.48 ± 0.016	0.20 ± 0.06	0.95 ± 0.022
**32b**	0.11 ± 0.033	0.17 ± 0.089	0.09 ± 0.005	0.16 ± 0.07
**32c**	0.50 ± 0.07	1.54 ± 0.49	0.37 ± 0.09	0.51 ± 0.041

## References

[1] Taylor R.D., MacCoss M., Lawson A.D.G. (2014). Rings in drugs. J. Med. Chem..

[2] Patel S.K., Patnayak S. (2024). Heterocyclic compounds an potential drug and its biological activity: A review. J. Nonlinear. Anal. Optim..

[3] Sharma P.K., Amin A., Kumar M. (2020). Synthetic Methods of Medicinally Important Heterocycles-thiazines: A Review. Open J. Med. Chem..

[4] Jampilek J. (2019). Heterocycles in Medicinal Chemistry. Molecules.

[5] Luo W., Liu Y., Qin H., Zhao Z., Wang S., He W., Tang S., Peng J. (2024). Nitrogen-containing heterocyclic drug products approved by the FDA in 2023: Synthesis and biological activity. Eur. J. Med. Chem..

[6] El-Garhy O.H. (2015). An overview of the azoles of interest. Int. J. Curr. Pharmaceut. Res..

[7] Thakur A., Verma M., Sharma R. (2022). Oxazole and isoxazole: From one-pot synthesis to medical applications. Tetrahedron.

[8] Wang Z., Xiong Y., Peng Y., Zhang X., Li S., Peng Y., Peng X., Zhuo L., Jiang W. (2023). Natural product evodiamine-inspired medicinal chemistry: Anticancer activity, structural optimization and structure-activity relationship. Eur. J. Med. Chem..

[9] Kumari G., Dhillon S., Rani P., Chahal M., Aneja D.K., Kinger M. (2024). Development in the Synthesis of Bioactive Thiazole-Based Heterocyclic Hybrids Utilizing Phenacyl Bromide. ACS Omega.

[10] Bérubé G. (2016). An overview of molecular hybrids in drug discovery. Expert Opin. Drug Discov..

[11] Gothelf K.V. (1996). Bis-Heterocyclic Derivatives. WIPO Patent.

[12] Bąchor U., Junka A., Brożyna M., Mączyński M. (2023). The In Vitro Impact of Isoxazole Derivatives on Pathogenic Biofilm and Cytotoxicity of Fibroblast Cell Line. Int. J. Mol. Sci..

[13] Kankala S., Kankala R.K., Gundepaka P., Thota N., Nerella S., Gangula M.R., Guguloth H., Kagga M., Vadde R., Vasam C.S. (2013). Regioselective synthesis of isoxazole–mercaptobenzimidazole hybrids and their in vivo analgesic and anti-inflammatory activity studies. Bioorg. Med. Chem. Lett..

[14] Mączyński M., Regiec A., Sochacka-Ćwikła A., Kochanowska I., Kocięba M., Zaczyńska E., Artym J., Kałas W., Zimecki M. (2021). Synthesis, Physicochemical Characteristics and Plausible Mechanism of Action of an Immunosuppressive Isoxazolo[5,4-e]-1,2,4-Triazepine Derivative (RM33). Pharmaceuticals.

[15] Mazlan M.K.N., Mohd Tazizi M.H.D., Ahmad R., Noh M.A.A., Bakhtiar A., Wahab H.A., Mohd Gazzali A. (2021). Antituberculosis Targeted Drug Delivery as a Potential Future Treatment Approach. Antibiotics.

[16] Lee Y.-S., Park S.M., Kim B.H. (2009). Synthesis of 5-isoxazol-5-yl-20-deoxyuridines exhibiting antiviral activity against HSV and several RNA viruses. Bioorg. Med. Chem. Lett..

[17] Bąchor U., Brożyna M., Junka A., Chmielarz M.R., Gorczyca D., Mączyński M. (2024). Novel Isoxazole-Based Antifungal Drug Candidates. Int. J. Mol. Sci..

[18] Müller-Schiffmann A., Sticht H., Korth C. (2012). Hybrid compounds: From simple combinations to nanomachines. BioDrugs.

[19] Sysak A., Obmińska-Mrukowicz B. (2017). Isoxazole ring as a useful scaffold in a search for new therapeutic agents. Eur. J. Med. Chem..

[20] Kakkar S., Narasimhan B. (2019). A comprehensive review on biological activities of oxazole derivatives. BMC Chem..

[21] Staderini M., Vanni S., Baldeschi A.C., Giachin G., Zattoni M., Celauro L., Ferracin C., Bistaffa E., Moda F., Pérez D.I. (2023). Bifunctional carbazole derivatives for simultaneous therapy and fluorescence imaging in prion disease murine cell models. Eur. J. Med. Chem..

[22] Matera C., Bono F., Pelucchi S., Collo G., Bontempi L., Gotti C., Zoli M., De Amici M., Missale C., Fiorentini C. (2019). The novel hybrid agonist HyNDA-1 targets the D3R-nAChR heteromeric complex in dopaminergic neurons. Biochem. Pharmacol..

[23] Fang L., Kraus B., Lehmann J., Heilmann J., Zhang Y., Decker M. (2008). Design and synthesis of tacrine-ferulic acid hybrids as multi-potent anti-Alzheimer drug candidates. Bioorg. Med. Chem. Lett..

[24] GBD 2021 Sickle Cell Disease Collaborators (2023). Global, regional, and national prevalence and mortality burden of sickle cell disease, 2000-2021: A systematic analysis from the Global Burden of Disease Study 2021. Lancet Haematol..

[25] Ting P.Y., Borikar S., Kerrigan J.R., Thomsen N.M., Aghania E., Hinman A.E., Reyes A., Pizzato N., Fodor B.D., Wu F. (2024). A molecular glue degrader of the WIZ transcription factor for fetal hemoglobin induction. Science.

[26] Bonazzi S., Cernijenko A., Stroka Cobb J., Dales N., Kerrigan J.R., Lam P., Malik H.A., O’brien G., Patterson A.W., Thomsen N.M.-F. (2021). Preparation of 3-(5-Methoxy-1-oxo-isoindolin-2-yl)piperidine-2,6-dione Derivatives for Treatment of Blood Disorders. WIPO Patent.

[27] Jumppanen M., Kinnunen S.M., Välimäki M.J., Talman V., Auno S., Bruun T., Boije af Gennäs G., Xhaard H., Aumüller I.B., Ruskoaho H. (2019). Synthesis, Identification, and Structure−Activity Relationship Analysis of GATA4 and NKX2-5 Protein−Protein Interaction Modulators. J. Med. Chem..

[28] Kinnunen S., Tölli M., Välimäki M., Jumppanen M., Boije af Gennäs G., Yli-Kauhaluoma J., Ruskoaho H. (2018). Isoxazole-Amides for Treating Cardiac Diseases. WIPO Patent.

[29] Välimäki M.J., Tölli M.A., Kinnunen S.M., Aro J., Serpi R., Pohjolainen L., Talman V., Poso A., Ruskoaho H.J. (2017). Discovery of Small Molecules Targeting the Synergy of Cardiac Transcription Factors GATA4 and NKX2-5. J. Med. Chem..

[30] Zemkova H. (2023). Purinergic P2 Receptors: Structure and Function 2.0. Int. J. Mol. Sci..

[31] Sutton J.C., Pi Z., Ruel R., L’heureux A., Thibeault C., Lam P.Y.S. (2006). 2-phenoxy-n- (1, 3, 4-thiadizol-2-yl) pyridin-3-amine Derivatives and Related Compounds as p2y1 Receptor Inhibitors for the Treatment of Thromboembolic Disorders. WIPO Patent.

[32] Haenisch B., Bönisch H. (2011). Depression and antidepressants: Insights from knockout of dopamine, serotonin or noradrenaline re-uptake transporters. Pharmacol. Ther..

[33] Ciraulo D.A., Oldham M. (2014). Sedative Hypnotics. The Effects of Drug Abuse on the Human Nervous System.

[34] Elmegeed G.A., Baiuomy A.R., Abdelhalim M.M., Hana H.Y. (2010). Synthesis and antidepressant evaluation of five novel heterocyclic tryptophan-hybrid derivatives. Arch. Pharm..

[35] Wang C., Wang Q., Ji B., Pan Y., Xu C., Cheng B., Bai B., Chen J. (2018). The Orexin/Receptor System: Molecular Mechanism and Therapeutic Potential for Neurological Diseases. Front. Mol. Neurosci..

[36] Branstetter B.J., Letavic M.A., Ly K.S., Rudolph D.A., Savall B.M., Shah C.R., Shireman B.T. (2011). Fused Heterocyclic Compounds as Orexin Receptor Modulators. WIPO Patent.

[37] Ghit A., Assal D., Al-Shami A.S., Eldin D., Hussein E. (2021). GABA_A_ receptors: Structure, function, pharmacology, and related disorders. J. Genet. Eng. Biotechnol..

[38] Cecere G., Zbinden K.G., Hernandez M.-C., Knust H., Koblet A., Olivares Morales M., Patiny-Adam A., Pinard E., Runtz-Schmitt V., Steiner S. (2019). New Isoxazolyl Ether Derivatives as Gaba a Alpha5 Pam. WIPO Patent.

[39] Skok M. (2022). Mitochondrial nicotinic acetylcholine receptors: Mechanisms of functioning and biological significance. Int. J. Biochem. Cell Biol..

[40] Crowley B.M., Campbell B.T., Chobanian H.R., Feels J.I., Guiadeen D.G., Greshock T.J., Leavitt K.J., Rada V.L., Bell I.M. (2019). Spiropiperidine Allosteric Modulators of Nicotinic Acetylcholine Receptors. WIPO Patent.

[41] Loix M., Vanherle S., Turri M., Kemp S., Fernandes K.J.L., Hendriks J.J.A., Bogie J.F.J. (2024). Stearoyl-CoA desaturase-1: A potential therapeutic target for neurological disorders. Mol. Neurodegener..

[42] Igal R.A., Sinner D.I. (2021). Stearoyl-CoA desaturase 5 (SCD5), a Δ-9 fatty acyl desaturase in search of a function. Biochim. Biophys. Acta Mol. Cell Biol. Lipids..

[43] Wrona I., Tivitmahaisoon P., Tardiff D., Pandya B., Ozboya K., Lucas M., Le Bourdonnec B. (2019). Compounds and Uses Thereof. WIPO Patent.

[44] Simonyi A., Schachtman T.R., Christoffersen G.R.J. (2010). Metabotropic glutamate receptor subtype 5 antagonism in learning and memory. Eur. J. Pharmacol..

[45] Burdi D., Spear K.L., Hardy L.W. (2010). Preparation of Substituted Oxazolopyridines and Their Analogs for Treating Disorders Mediated by Metabotropic Glutamate Receptor 5.

[46] Ishak A., Mazonakis N., Spernovasilis N., Akinosoglou K., Tsioutis C. (2025). Bactericidal versus bacteriostatic antibacterials: Clinical significance, differences and synergistic potential in clinical practice. J. Antimicrob. Chemother..

[47] Williams T.L., Yin Y.W., Carter C.W. (2016). Selective Inhibition of Bacterial Tryptophanyl-tRNA Synthetases by Indolmycin Is Mechanism-based. J. Biol. Chem..

[48] Barvian K., Basarab G.S., Gowravaram M.R., Hauck S.I., Zhou F. (2010). Fused, Spirocyclic Heteroaromatic Compounds for the Treatment of Bacterial Infections. WIPO Patent.

[49] Best D.J., Elder J.S., Osborne N.F. (1997). Preparation of Sulfamoyl-Containing Alditols as Bactericides and t-RNA Synthetase Inhibitors. WIPO Patent.

[50] Broom N.J.P., Elder J.S., Hannan P.C.T., Pons J.E., O’Hanlon P.J., Walker G., Wilson J., Woodall P. (1995). The chemistry of pseudomonic acid. Part 14. Synthesis and in vivo biological activity of heterocycle substituted oxazole derivatives. J. Antibiot..

[51] Wales S.M., Hammer K.A., Somphol K., Kemker I., Schroder D.C., Tague A.J., Brkic Z., King A.M., Lyras D., Riley T.V. (2015). Synthesis and antimicrobial activity of binaphthyl-based, functionalized oxazole and thiazole peptidomimetics. Org. Biomol. Chem..

[52] Becker D., Selbach M., Rollenhagen C., Ballmaier M., Meyer T.F., Mann M., Bumann D. (2006). Robust Salmonella metabolism limits possibilities for new antimicrobials. Nature.

[53] Magalhães J., Franko N., Raboni S., Annunziato G., Tammela P., Bruno A., Bettati S., Armao S., Spadini C., Cabassi C.S. (2021). Discovery of substituted (2-aminooxazol-4-yl)isoxazole-3-carboxylic acids as inhibitors of bacterial serine acetyltransferase in the quest for novel potential antibacterial adjuvants. Pharmaceuticals.

[54] Azzali E., Girardini M., Annunziato G., Pavone M., Vacondio F., Mori G., Rosalia M.P., Constantino G., Pieroni M. (2020). 2-Aminooxazole as a Novel Privileged Scaffold in Antitubercular Medicinal Chemistry. ACS Med. Chem. Lett..

[55] Lilienkampf A., Pieroni M., Wan B., Wang Y., Franzblau S.G., Kozikowski A.P. (2010). Rational design of 5-phenyl-3-isoxazolecarboxylic acid ethyl esters as growth inhibitors of *Mycobacterium tuberculosis*. a potent and selective series for further drug development. J. Med. Chem..

[56] Lilienkampf A., Pieroni M., Franzblau S.G., Bishai W.R., Kozikowski A.P. (2012). Derivatives of 3-isoxazolecarboxylic acid esters: A potent and selective compound class against replicating and nonreplicating *Mycobacterium tuberculosis*. Curr. Top. Med. Chem..

[57] Girardini M., Ferlenghi F., Annunziato G., Degiacomi G., Papotti B., Marchi C., Sammartino J.C., Rasheed S.S., Contini A., Pasca M.R. (2023). Expanding the knowledge around antitubercular 5-(2-aminothiazol-4-yl)isoxazole-3-carboxamides: Hit-to-lead optimization and release of a novel antitubercular chemotype via scaffold derivatization. Eur. J. Med. Chem..

[58] International Agency for Research on Cancer. https://www.iarc.who.int/wp-content/uploads/2024/02/pr345_E.pdf.

[59] Baier A., Szyszka R. (2020). Compounds from Natural Sources as Protein Kinase Inhibitors. Biomolecules.

[60] Cui J., Bhumralkar D., Botrous I., Chu J.Y., Funk L.A., Hanau C.E., Harris G.D., Jia L., Johnson J., Kolodziej S.A. (2004). Aminoheteroaryl Compounds as Protein Kinase Inhibitors. WIPO Patent.

[61] Anbalagan M., Rowan B.G. (2015). Estrogen receptor alpha phosphorylation and its functional impact in human breast cancer. Mol. Cell Endocrinol..

[62] Dijcks F.A., Lusher S.J., Stock H.T., Veeneman G.H. (2012). N-Substituted Azetidine Derivatives. WIPO Patent.

[63] Pohanka M. (2012). Alpha7 Nicotinic Acetylcholine Receptor Is a Target in Pharmacology and Toxicology. Int. J. Mol. Sci..

[64] Zavoronkovs A., Aliper A., Aladinskiy V., Kukharenko A., Qin L., Cheng X. (2022). Preparation of Imidazole Analogs as TNIK and MAP4K4 Kinases Inhibitors for the Treatment of TNIK-Mediated Diseases. WIPO Patent.

[65] Qian S., Wei Z., Yang W., Huang J., Yang Y., Wang J. (2022). The role of BCL-2 family proteins in regulating apoptosis and cancer therapy. Front. Oncol..

[66] Sochacka-Ćwikła A., Regiec A., Zimecki M., Artym J., Zaczyńska E., Kocięba M., Kochanowska I., Bryndal I., Pyra A., Mączyński M. (2020). Synthesis and Biological Activity of New 7-Amino-oxazolo[5,4-*d*]Pyrimidine Derivatives. Molecules.

[67] Sochacka-Ćwikła A., Mączyński M., Czyżnikowska Ż., Wiatrak B., Jęśkowiak I., Czerski A., Regiec A. (2022). New oxazolo[5,4-*d*]pyrimidines as potential anticancer agents: Their design, synthesis, and in vitro biological activity research. Int. J. Mol. Sci..

[68] Wang X., Bove A.M., Simone G., Ma B. (2020). Molecular Bases of VEGFR-2-Mediated Physiological Function and Pathological Role. Front. Cell Dev. Biol..

[69] Sochacka-Ćwikła A., Regiec A., Czyżnikowska Ż., Śliwińska-Hill U., Kwiecień A., Wiatrak B., Rusak A., Krawczyńska K., Mrozowska M., Borska S. (2024). Synthesis and structural proof of novel oxazolo[5,4-*d*]pyrimidine derivatives as potential VEGFR2 inhibitors. in vitro study of their anticancer activity. Bioorg. Chem..

[70] Kim D., Nam H.J. (2022). PARP Inhibitors: Clinical Limitations and Recent Attempts to Overcome Them. Int. J. Mol. Sci..

[71] Hawkins N. (2011). Phthalazinone Compound as Parp Inhibitor. WIPO Patent.

[72] Jung J., Kwon J., Hong S., Moon A.-N., Jeong J., Kwon S., Kim J., Lee M., Lee H., Lee J.H. (2020). Discovery of novel heat shock protein (Hsp90) inhibitors based on luminespib with potent antitumor activity. Bioorg. Med. Chem. Lett..

[73] Kang J.-H., Lee H.-S., Kwon J.-S., Park J.-T., Hong C.-S., Shin D.-H., Hong S.-J., Moon A.-N., Jeong J.-A., Kwon S.-W. (2011). A Novel 5-Membered Heterocycle Derivatives and Manufacturing Process Thereof. WIPO Patent.

[74] Chen Y.K., Nie D.T. (2009). Pregnane X receptor and its potential role in drug resistance in cancer treatment. Recent Pat. Anti-Cancer Drug Discov..

[75] Hodnik Ž., Maši L.P., Tomaši T., Smodiš D., D’Amore C., Fiorucci S., Kikelj D. (2014). Bazedoxifene scaffold-based mimetics of solomonsterols A and B as novel pregnane X receptor antagonists. J. Med. Chem..

[76] Premalatha S., Rambabu G., Hatti I., Ramachandran D. (2020). Design, Synthesis and Biological Evaluation of 3-(3,4,5-Trimethoxyphenyl)-5-(2-(5-arylbenzo[b]thiophen-3-yl)oxazol-5-yl)isoxazole Derivatives as Anticancer Agents. Lett. Org. Chem..

[77] Jandl K., Heinemann A. (2017). The therapeutic potential of CRTH2/DP2 beyond allergy and asthma. Prostaglandins Other Lipid Mediat..

[78] Xiao D., Zhu X., Yu Y., Shao N., Wu J., McCormick K.D., Dhondi P., Qin J., Mazzola R., Tang H. (2014). Quality by design (QbD) of amide isosteres: 5,5-Disubstituted isoxazolines as potent CRTh antagonists with favorable pharmacokinetic and drug-like properties. Bioorg. Med. Chem. Lett..

[79] Aslanian R.G., Boyce C.W., Mazzola R.D., Mckittrick B.A., Mccormick K.D., Palani A., Qin J., Tang H., Xiao D., Yu Y. (2012). Preparation of Quinazolinone Compounds as CRTH2 Antagonists. WIPO Patent.

[80] Canovas B., Nebreda A.R. (2021). Diversity and versatility of p38 kinase signalling in health and disease. Nat. Rev. Mol. Cell Biol..

[81] Severance D.L., Gardiner E.M.M., Noble S.A., Lou B., Borchardt A.J., Kahraman M., Roppe J.R., Siegel D.L., Scranton S.A. (2006). Heterocyclic Ortho-Terphenyl Analogs (Thiazoles, Oxazoles, Isoxazoles, and Pyrazoles, etc.) as Inhibitors of p38 Kinase, and Methods of Treating Inflammatory Disorders and Other Diseases Using Them.

[82] Zenobia C., Hajishengallis G. (2015). Basic biology and role of interleukin-17 in immunity and inflammation. Periodontol. 2000.

[83] Tau G., Rothman P. (1999). Biologic functions of the IFN-gamma receptors. Allergy.

[84] Leban J., Baumgartner R., Saeb W., Chevrier C. (2012). Preparation of Pyrazolylisoxazoles as IL-17 and IFN-gamma Production Inhibitors for Treating Autoimmune Inflammation.

[85] Leban J., Baumgartner R., Saeb W., Chevrier C. (2012). Preparation of Pyrazolylisoxazoles as IL-17 and IFN-gamma Production Inhibitors for Treating Autoimmune Inflammation.

[86] Cohen P., Snelling T. (2025). Diseases caused by altered specificity of a protein kinase for its allosteric activators. Trends Biochem. Sci..

[87] Du N., Li Z., Wang H., Tian Z., Ji Z., Chen Y., O’Yang C. (2024). Preparation of (hetero)aryl carboxamides as Novel ALPK1 Inhibitors.

[88] Zhang H., Zhou X.D., Shapiro M.D., Lip G.Y.H., Tilg H., Valenti L., Somers V.K., Byrne C.D., Targher G., Yang W. (2024). Global burden of metabolic diseases, 1990–2021. Metabolism.

[89] Varga T., Czimmerer Z., Nagy L. (2011). PPARs are a unique set of fatty acid regulated transcription factors controlling both lipid metabolism and inflammation. Biochim. Biophys. Acta.

[90] Lowe D.B., Bifulco N., Bullock W.H., Claus T., Coish P., Dai M., Dela Cruz F.E., Dickson D., Fan D., Hoover-Litty H. (2006). Substituted indanylacetic acids as PPAR-α-γ activators. Bioorg. Med. Chem. Lett..

[91] Ji G., Guo Q., Xue Q., Kong R., Wang S., Lei K., Liu R., Wang X. (2021). Novel GPR120 Agonists with Improved Pharmacokinetic Profiles for the Treatment of Type 2 Diabetes. Molecules.

[92] Hirasawa A., Tsumaya K., Awaji T., Katsuma S., Adachi T., Yamada M., Sugimoto Y., Miyazaki S., Tsujimoto G. (2005). Free fatty acids regulate gut incretin glucagon-like peptide-1 secretion through GPR120. Nat. Med..

[93] Ma J., Novack A., Nashashibi I., Pham P., Rabbat C.J., Song J., Shi D.F., Zhao Z., Choi Y.-J., Chen X. (2010). Aryl gpr120 Receptor Agonists and Uses Thereof. WIPO Patent.

[94] Octave M., Pirotton L., Ginion A., Robaux V., Lepropre S., Ambroise J., Bouzin C., Guigas B., Giera M., Foretz M. (2021). Acetyl-CoA Carboxylase Inhibitor CP640.186 Increases Tubulin Acetylation and Impairs Thrombin-Induced Platelet Aggregation. Int. J. Mol. Sci..

[95] Yasuma T., Kamata M., Yamashita T., Hirose H., Murakami M., Kina A., Yonemori K., Mizojiri R., Fujimori I., Fujimoto T. (2012). Preparation of Heterocyclic Bicyclic Compounds as Acetyl-CoA Carboxylase Inhibitors. WIPO Patent.

[96] Nishikimi M., Choudhary R.C., Shoaib M., Yagi T., Becker L.B., Kim J. (2023). Neurological Improvement via Lysophosphatidic Acid Administration in a Rodent Model of Cardiac Arrest-Induced Brain Injury. Int. J. Mol. Sci..

[97] Buckman B.O., Nicholas J.B., Emayan K., Seiwert S.D. (2013). Preparation of N-Heterocyclylcarbamates as Lysophosphatidic Acid (LPA) Receptor Antagonists. WIPO Patent.

[98] Weaver A.K., Bomben V.C., Sontheimer H. (2006). Expression and function of calcium-activated potassium channels in human glioma cells. Glia.

[99] Hongu M., Hosaka T., Kashiwagi T., Kono R., Kobayashi H. (2002). Preparation of Substituted Imidazoles/Oxazoles/Thiazoles as Large Conductance Calcium-Activated K Channel Openers. WIPO Patent.

[100] Grieco M., De Caris M.G., Maggi E., Armeli F., Coccurello R., Bisogno T., D’Erme M., Maccarrone M., Mancini P., Businaro R. (2021). Fatty Acid Amide Hydrolase (FAAH) Inhibition Modulates Amyloid-Beta-Induced Microglia Polarization. Int. J. Mol. Sci..

[101] Genovese T., Duranti A., D’Amico R., Fusco R., Impellizzeri D., Peritore A.F., Crupi R., Gugliandolo E., Cuzzocrea S., Di Paola R. (2022). Fatty Acid Amide Hydrolase (FAAH) Inhibition Plays a Key Role in Counteracting Acute Lung Injury. Int. J. Mol. Sci..

[102] Chobanian H., Lin L.S., Liu P., Chioda M.D., Devita R.J., Nargund R.P., Guo Y., Hamill T., Li W., Henze D.A. (2011). Preparation of Pyridinylsulfanyloxazole Derivatives and Analogs for Use as FAAH Inhibitors. WIPO Patent.

[103] Chen S., Wu L., Lang B., Zhao G., Zhang W. (2025). Sphingosine 1-phosphate receptor 1 modulators exert neuroprotective effects in central nervous system disorders. Front. Pharmacol..

[104] Coyle P.K., Freedman M.S., Cohen B.A., Cree B.A.C., Markowitz C.E. (2024). Sphingosine 1-phosphate receptor modulators in multiple sclerosis treatment: A practical review. Ann. Clin. Transl. Neurol..

[105] Murali Dhar T.G., Xiao H.-Y., Watterson S.H., Ko S.S., Dyckman A.J., Langevine C.M., Das J., Cherney R.J. (2011). Tricyclic Heterocyclic Compounds. WIPO Patent.

[106] Xiao H.-Y., Watterson S.H., Langevine C.M., Srivastava A.S. (2016). Identification of Tricyclic Agonists of Sphingosine-1-Phosphate Receptor 1 (S1P1) Employing Ligand-Based Drug Design. J. Med. Chem..

[107] Moon D.O. (2025). Review of Cathepsin K Inhibitor Development and the Potential Role of Phytochemicals. Molecules.

[108] Barrett D.G., Catalano J.G., Deaton D.N., Miller A.B., Ray J.A., Samano V. (2003). Derivatives of 1-(Oxoaminoacetyl) Pentylcarbamate as Cathepsin k Inhibitors for the Treatment of Bone Loss. WIPO Patent.

[109] Kim H., Ronai Z.A. (2020). PRMT5 function and targeting in cancer. Cell Stress..

[110] Machacek M., Altman M.D., Kawamura S., Reutershan M.H., Sloman D.L., Siliphaivanh P., Schneider S.E., Yeung C.S., Witter D.J., Gibeau C.R. (2021). Spiro-Isoquinoline-3,4’-Piperidine Derivatives as PRMT5 Inhibitors and Their Preparation. WIPO Patent.

[111] Jetten A.M., Takeda Y., Slominski A., Kang H.S. (2018). Retinoic acid-related Orphan Receptor γ (RORγ): Connecting sterol metabolism to regulation of the immune system and autoimmune disease. Curr. Opin. Toxicol..

[112] Qiu R., Wang Y. (2018). Retinoic Acid Receptor-Related Orphan Receptor γt (RORγt) Agonists as Potential Small Molecule Therapeutics for Cancer Immunotherapy. J. Med. Chem..

[113] Kotoku M., Takaki M., Noriyosh S., Shintaro H., Shingo F., Shingo O., Hiroshi Y., Masahiro Y., Takayuki S., Kazuyuki H. (2014). Preparation of Isoxazoles and Their Use as ROR-γ Antagonists and Pharmaceutical. WIPO Patent.

[114] Kotoku M., Maeba T., Fujioka S., Yokota M., Seki N., Ito K., Suwa Y., Ikenogami T., Hirata K., Hase Y. (2019). Discovery of Second Generation RORγ Inhibitors Composed of an Azole Scaffold. J. Med. Chem..

[115] Baloglu E., Bohnert G.J., Ghosh S., Lobera M., Schmidt D.R., Sung L. (2013). Preparation of Phenylphenylmethylisoxazolylbenzofuranylactamide Derivatives and Analogs for Use as Retinoid-Related Orphan Receptor Gamma Modulators.

[116] Morais T.S. (2024). Recent Advances in the Development of Hybrid Drugs. Pharmaceutics.

